# Trainee Awareness of US Preventive Services Task Force (USPSTF) Colorectal Cancer Screening Guidelines

**DOI:** 10.1007/s13187-025-02638-7

**Published:** 2025-05-06

**Authors:** Madaliene Denison, Jinman Cai, Hannah H. Roberson, Ellen Lockhart, Farrell Adkins

**Affiliations:** 1 Department of Surgery, Carilion Clinic, Virginia Tech Carilion School of Medicine, 1906 Belleview Ave SE, Roanoke, VA 24014 USA; 2 Health Analytics Research, Carilion Clinic, Roanoke, VA 24014 USA

**Keywords:** Colorectal cancer screening, Resident education, Colorectal cancer, Screening guidelines

## Abstract

In 2021, the US Preventive Services Task Force (USPSTF) issued a final recommendation to lower the initial age for colorectal cancer (CRC) screening to 45^1^. At many institutions, trainees play a critical role in the implementation of CRC screening guidelines. The aim of the current study is to assess trainee awareness of recent changes to CRC screening guidelines. Trainees in surgical and non-surgical residency and fellowship programs at a single academic medical center were surveyed, in February of 2023, regarding awareness of recent changes to CRC screening guidelines. Additional information regarding trainee recommendations for various types of screening modalities and their perceptions of patient awareness was obtained. A total of 116 trainees responded to the survey, reflecting a 39% response rate. Of trainees recommending CRC screening, 75% recommended screening to be initiated at age 45, 13% at age 50, 6% at age 40, and 2% at age greater than 50. Trainee knowledge of screening guidelines came from medical school learning (70.7%) and USPSTF guidelines (82%). The most recommended screening modalities were colonoscopy (87%), fecal immunochemical testing (FIT) (31%), and fecal occult blood testing (FOBT) (15%). In comparing trainees who perform colonoscopies versus trainees from primary care specialties, there was no statistical difference between groups regarding knowledge on current screening guideline recommendations. Most trainees recommended CRC screening during their training. Unfortunately, many trainees were unaware of recent changes to CRC screening guidelines. These results revealed an important knowledge gap to be addressed among surgical and non-surgical trainees.

## Introduction

The incidence of colorectal cancer (CRC) in younger individuals has been steadily rising, requiring enhanced screening efforts for early detection and intervention. In 1995, the proportion of reported CRC cases among those under the age of 55 was 11%, which has increased to 20% in 2019 [1]. Increasing rates of obesity nationally are one possible explanation for the increase in cases in young adults [2]. The US Preventive Services Task Force (USPSTF) decreased the age of screening for asymptomatic patients with average CRC risk from 50 to 45 in 2021 in concordance with the increased incidence of CRC in younger adults [1]. Despite the availability of comprehensive guidelines, previous studies have suggested a gap between recommendations and application in clinical practice [3, 4]. More specifically, resident physicians have been found to have decreased knowledge and understanding of colorectal cancer screening guidelines compared to attending physicians [3]. Resident physicians uniquely represent a cohort at the intersection of medical education and clinical application. Understanding the extent of their knowledge, adherence to guidelines, and potential barriers to application of guidelines is crucial for addressing disparities in colorectal cancer screening rates and appropriate follow up.

Studies have differed on if resident knowledge increases as they progress through training. Genneralli et al. conducted a survey involving resident and attending physicians on screening guidelines for CRC, revealing an increase in knowledge base with additional years of education [3]. Conversely, a study by Willett et al. reviewed health care screening outcomes of patients cared for by post-graduate year (PGY) 1 versus post-graduate year 2 resident physicians. This study demonstrated no difference in breast and colorectal cancer screening outcomes between the two groups, suggesting little variation between screening knowledge base and years of residency training [5]. Multiple other studies have demonstrated that PGY level in internal medicine does not affect the rate of CRC screening or adherence to screening guidelines [6, 7]. Upon further review, Akerman et al. discovered that residents are knowledgeable about CRC screening guidelines for colonoscopy with average risk individuals but that they are less familiar with fecal occult blood testing (FOBT) guidelines and other screening options [8]*.* As demonstrated, a myriad of studies, spanning a 20-year period, highlight persistent knowledge-based discrepancies in residents’ adherence to CRC screening guidelines [3, 5–8]. This investigation’s main objective is to evaluate the level of awareness among general surgery resident physicians regarding the newly revised US Preventive Services Task Force (USPSTF) colon and rectal cancer screening guidelines.

## Methods

A 15-item survey was designed to assess the awareness and application of recently updated USPSTF guidelines on colorectal cancer screening (Appendixes 1 and 2). Following institutional review board (IRB) approval, the survey was administered to all residents and fellow physicians, within specialties who regularly recommend colorectal cancer screening, employed at a single academic medical center in February of 2023. These specialties included general surgery, obstetrics and gynecology, internal medicine, family medicine, and gastroenterology. Six questions were related to demographic information. The remaining questions focused on individual practice patterns related to the recommended age to commence CRC screening, including primary sources of screening guideline information, most utilized types of screening, and follow-up.

The data was collected and managed using Research Electronic Data Capture (REDCap) tools [9, 10]. REDCap is a secure, web-based software platform designed to support data capture for research studies. REDCap at our institution is housed in the Health Analytics and Research department, and the tool provides (1) an intuitive interface for validated data capture, (2) audit trails for tracking data manipulation and export procedures, (3) automated export procedures for seamless data downloads to common statistical packages, and (4) procedures for data integration and interoperability with external sources.

The biostatistician on our research team served as the honest broker for the data to keep clinical members of the team blinded to individual responses. Through REDCap, 336 survey invitations were e-mailed to over 25 specialties and subspecialties at our institution. Survey links in the e-mails were personal to the recipient and were not to be shared with other individuals. After the initial survey invitation was launched, invited physicians who had not yet responded to the survey were sent automated reminders once every 3 days up to three times until either they responded or the survey closed. Data was downloaded for cleaning from REDCap into Microsoft Excel, after which they were transferred to SAS Enterprise Guide 8.3 (64-bit) for analysis.

## Results

A total of 300 residents and fellows were invited to participate in the study, with a response rate of 34.5% (116 participants). Respondents included 31 PGY-1, 31 PGY-2, 27 PGY-3, 16 PGY-4, 9 PGY-5, and 2 PGY-6 trainees. Among participants, 70.2% reported routinely recommending colorectal cancer screening in their clinical practice. Notably, 80% of respondents identified as “non-proceduralists,” representing specialties such as family medicine, internal medicine, and obstetrics and gynecology.

**Fig. 1:**
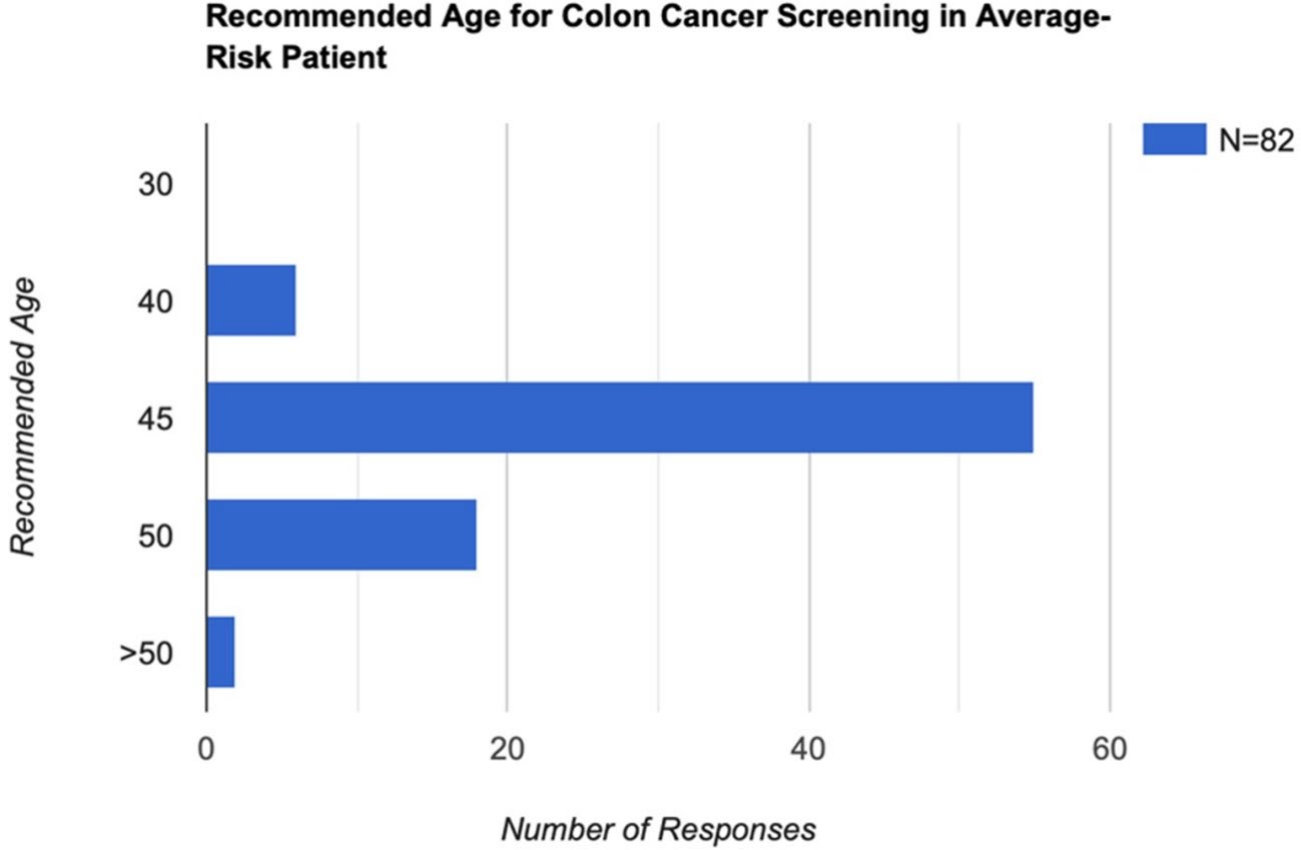
Recommended age for colon cancer screening in average-risk patient

When asked about the appropriate age to initiate CRC screening in average-risk patients, 70% of respondents correctly identified age 45, in alignment with current guidelines; the remaining 30% of respondents provided responses that deviated from these recommendations (Fig. 1). Stratified by postgraduate year, the proportion of correct responses was as follows: PGY-1, 25 of 31 (80.6%); PGY-2, 20 of 31 (64.5%); PGY-3, 16 of 27 (59.3%); PGY-4, 8 of 16 (50.0%); PGY-5, 7 of 9 (77.8%); and PGY-6, 1 of 2 (50.0%).

**Fig. 2:**
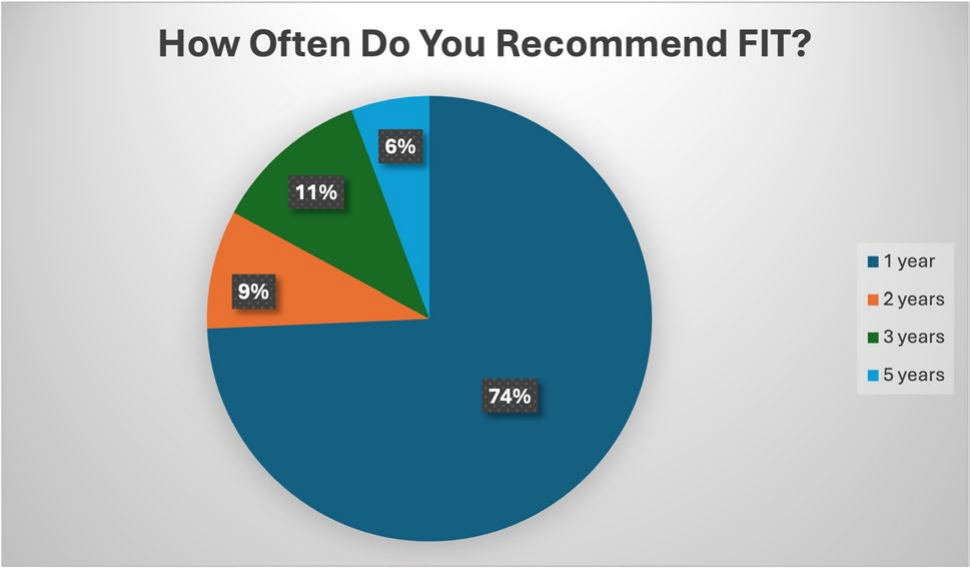
Recommended screening modalities using FIT

**Fig. 3:**
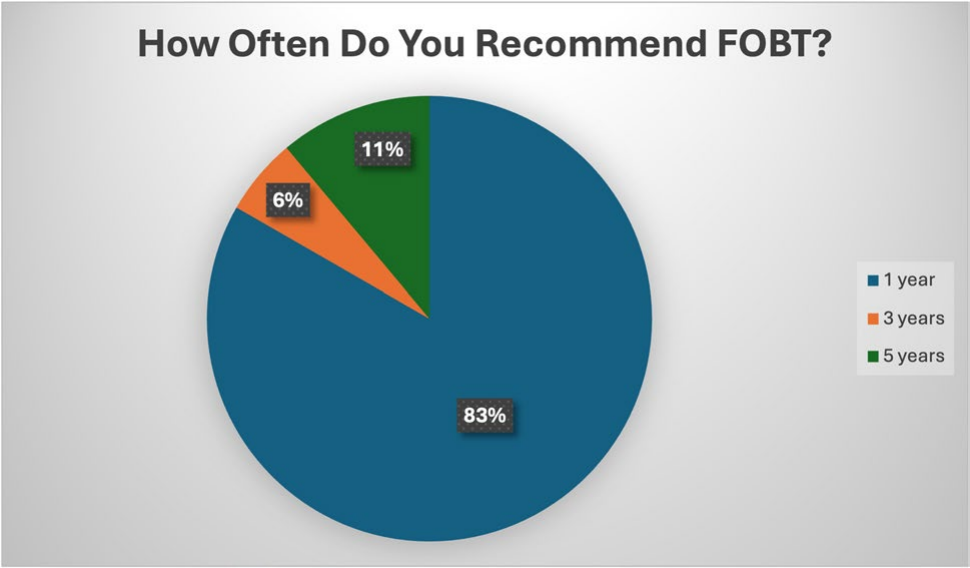
Recommended screening modalities using FOBT

Concerning their primary sources of screening guideline information, responders identified their medical school knowledge (73.3%) and USPSTF guidelines (70.4%) as their main sources. Other responses included health maintenance recommendations through the electronic medical record (17.2%) and subspeciality guidelines (26.7%). Regarding the screening modalities most recommended by trainees, most responders identified colonoscopy (87%), and some also reported utilizing FOBT (15.5%) or fecal immunochemical testing (FIT) (31%). 74.3% of FIT users (Fig. 2) and 83.3% of FOBT users (Fig. 3) demonstrated follow-up recommendations consistent with USPSTF guidelines. Most residents felt that patients understood the importance of CRC screening, but a proportion felt that patients did not.

## Discussion

The current study delves into the landscape of resident physician knowledge and practices concerning colorectal cancer screening guidelines, providing valuable insights. The primary focus of our investigation was to assess awareness of the recently updated USPSTF screening guidelines for colorectal cancer. While a majority of responders appropriately identified the recommended age to commence screening in average risk individuals (age 45), approximately 30% still inaccurately identified the age, revealing persistent knowledge deficits across trainees. Delayed screening could lead to worse outcomes, including higher healthcare costs related to more aggressive treatment in the setting of more advanced disease and increased mortality. A study by Abdelsattar et al. demonstrated that patients diagnosed with CRC before the age of 50 presented with more advanced disease than those diagnosed after the age of 50 [11].

The persistent knowledge deficit demonstrated in the current study reiterates broader issues within healthcare systems where screening guideline adherence remains problematic. The current findings echo previous reports that despite a foundational understanding of CRC screening modalities among residents, gaps persist in areas including risk factors, appropriate screening initiation ages, and the distinction between screening and prevention [7]. The current study similarly identifies continued knowledge gaps, which may potentiate suboptimal screening practices and decreasing early detection opportunities.

Studies on residents’ knowledge and adherence to CRC screening and surveillance guidelines indicated that, while knowledge improves as trainees advance through their years, senior residents did not consistently perform better than junior residents [3, 5–8]. This is also echoed in our study where post graduate year was not associated with improved knowledge of screening guidelines and modalities. Another study evaluating obstetrics and gynecology residents found that while most respondents were aware of CRC guidelines, significant gaps remained in applying them in clinical practices [12]. Taken together, these studies emphasize the variability in CRC screening knowledge and practices across physician trainees and the importance of focused education on improving practice, regardless of post graduate year.

Appropriate follow-up intervals for non-invasive screening modalities are also a key area for potential education. With the ease of at home testing like FOBT/FIT, physicians must understand the availability of this type of testing and appropriate follow-up. As demonstrated in our study, only 30 of the 82 residents who regularly recommend colorectal cancer screening utilized non-invasive testing. A thorough understanding of the limitations of non-invasive modalities is key to understanding the best opportunities to implement their use.

This study has some limitations. Given that the survey was provided at a single academic center, it has poor generalizability. The study can be expanded to include other academic centers as well as community centers to increase generalizability. While sample size for this study was reasonable, response rate was only 34.5%. A stronger study would include higher response rate and larger sample size for a more accurate representation. Also, surveys inherently create an environment for response bias. This survey attempted to limit response bias and subjective responses by providing multiple choice answers.

Education interventions geared toward improving resident knowledge and screening guidelines will be important to improving awareness among trainees. Khan et al. highlighted the potential for knowledge enhancement through a smartphone application [13]. The application was created as a simple tool for residents to access when they had questions about CRC guidelines, providing information about each screening tool, when it should be implemented, and follow up recommendations associated with individual tools [13].

Furthermore, a study on internal medicine residents’ practices in screening high-risk populations, specifically African American patients, for CRC highlighted a concerning trend: residents were inconsistent in performing and recommending screenings exams, which could be attributed to knowledge gaps and limited resources. This study assessed the effectiveness of educational intervention of a formal lecture focused on racial disparities in CRC and found statistically significant improvement in resident performance on endoscopic exams post intervention, although there were no significant changes in rectal exams or fecal occult blood testing [14]. This suggests that targeted education may enhance the performance of endoscopic procedures, though further efforts are needed to continue to improve adherence to CRC screening guidelines.

Another study completed at a university center implemented a low-cost educational health maintenance card, created by medical residents, to improve adherence to cancer screening guidelines. After implementation, adherence to appropriate screening significantly increased for cervical (40.8%), breast (33.2%), and colorectal cancer (20.5%) among average-risk patients. Inappropriate screening also notably decreased for cervical (26.8%) and breast cancer (32.8%), with minimal change for colorectal cancer. The intervention led to an estimated annual savings of nearly $1 million by reducing unnecessary testing. Additionally, resident knowledge of screening guidelines improved significantly and remained elevated 2 years post-intervention. The findings support that a simple, low-cost tool can enhance guideline adherence, reduce unnecessary screening, and generate meaningful cost savings [15].

Additionally, while artificial intelligence (AI) tools like ChatGPT show potential in assisting with CRC screening guidelines and surveillance, their performance in providing accurate, guideline-based recommendations remains limited. This study evaluated ChatGPT-3.5’s ability to provide accurate colorectal cancer screening and surveillance recommendations using 10 clinical vignettes, comparing its performance to that of expert physicians with and without a CRC mobile application. ChatGPT’s average accuracy was 45%, significantly lower than physicians without the application (56%) and those using the application (77%) it also demonstrated inconsistencies across sessions. The authors concluded that while AI tools like ChatGPT could have future potential, current versions lack the accuracy and consistency and are not recommended for guideline recommendations [16].

Our institution remains committed to enhancing guideline-specific colorectal cancer screening and surveillance recommendations. However, this continues to be a challenge due to the need for consistent education across multiple specialties at the institutional level. With the advancement of mobile applications, future artificial intelligence, and improved resident educational lectures, we hope to utilize these tools to support consistent, evidence-based practices across departments.

## Conclusion

In conclusion, while the current study demonstrates that most residents adhere to colorectal cancer screening guidelines, it also identifies specific areas where targeted educational interventions may enhance knowledge and ensure standardization of CRC screening practices. Efforts to streamline education, particularly regarding age-specific recommendations and follow-up protocols for alternative screening modalities, are essential for optimizing CRC screening practices among the future generation of healthcare professionals.

## Data Availability

Data is available upon request from authors.

## Abbreviations

CRC: Colorectal cancer
USPSTF: US Preventive Services Task Force
PGY: Post-graduate year
FOBT: Fecal occult blood testing
REDCap: Research Electronic Data Capture
FIT: Fecal immunochemical testing
AI: Artificial intelligence
